# Lipopolysaccharide and Recombinant Prion Protein Induce Distinct Neurodegenerative Pathologies in FVB/N Mice

**DOI:** 10.3390/ijms26136245

**Published:** 2025-06-28

**Authors:** Seyed Ali Goldansaz, Dagnachew Hailemariam, Elda Dervishi, Grzegorz Zwierzchowski, Roman Wójcik, David S. Wishart, Burim N. Ametaj

**Affiliations:** 1 Department of Agricultural, Food and Nutritional Science, University of Alberta, Edmonton, AB T6G 2P5, Canada; goldansaz@ualberta.ca (S.A.G.); dervishi@ualberta.ca (E.D.); grzegorz.zwierzchowski@uwm.edu.pl (G.Z.); 2 Sustainable Livestock Systems Branch, Sustainable Agri-Food Sciences Division, Agri-Food & Biosciences Institute, Hillsborough BT26 6DR, UK; 3 Faculty of Biology and Biotechnology, University of Warmia and Mazury, 1a Oczapowskiego Str., 10-719 Olsztyn, Poland; 4 Faculty of Veterinary Medicine, University of Warmia and Mazury, 1a Oczapowskiego Str., 10-719 Olsztyn, Poland; brandy@uwm.edu.pl; 5 Department of Biological Sciences, University of Alberta, Edmonton, AB T6G 2E9, Canada; david.wishart@ualberta.ca; 6 Department of Computing Science, University of Alberta, Edmonton, AB T6G 2E9, Canada

**Keywords:** recombinant prion protein, lipopolysaccharide, cofactor, neurodegeneration, prion disease

## Abstract

Prion diseases are classically attributed to the accumulation of protease-resistant prion protein (PrP^Sc^); however, recent evidence suggests that alternative misfolded prion conformers and systemic inflammatory factors may also contribute to neurodegeneration. This study investigated whether recombinant moPrP^Res^, generated by incubating wild-type mouse PrP^C^ with bacterial lipopolysaccharide (LPS), can induce prion-like disease in FVB/N female mice, whether LPS alone causes neurodegeneration, and how LPS modulates disease progression in mice inoculated with the Rocky Mountain Laboratory (RML) strain of prions. Wild-type female FVB/N mice were randomized into six subcutaneous treatment groups: saline, LPS, moPrP^Res^, moPrP^Res^ + LPS, RML, and RML + LPS. Animals were monitored longitudinally for survival, body weight, and clinical signs. Brain tissues were analyzed histologically and immunohistochemically for vacuolar degeneration, PrP^Sc^ accumulation, reactive astrogliosis, and amyloid-β plaque deposition. Recombinant moPrP^Res^ induced a progressive spongiform encephalopathy characterized by widespread vacuolation and astrogliosis, yet with no detectable PrP^Sc^ by Western blot or immunohistochemistry. LPS alone triggered a distinct neurodegenerative phenotype, including cerebellar amyloid-β plaque accumulation and terminal-stage spongiosis, with approximately 40% mortality by the end of the study. Co-administration of moPrP^Res^ and LPS resulted in variable regional pathology and intermediate survival (50% at 750 days post-inoculation). Interestingly, RML + LPS co-treatment led to earlier clinical onset and mortality compared to RML alone; however, vacuolation levels were not significantly elevated and, in some brain regions, were reduced. These results demonstrate that chronic endotoxemia and non-infectious misfolded PrP conformers can independently or synergistically induce key neuropathological hallmarks of prion disease, even in the absence of classical PrP^Sc^. Targeting inflammatory signaling and toxic prion intermediates may offer novel therapeutic strategies for prion and prion-like disorders.

## 1. Introduction

Prion diseases, also referred to as transmissible spongiform encephalopathies (TSEs), occupy a unique position among neurodegenerative conditions due to their infectious nature and the central role played by misfolded scrapie prion protein (PrP^Sc^) [1,2]. According to the widely accepted “protein-only” hypothesis, prion pathogenesis arises when the normally soluble cellular prion protein (PrP^C^) adopts a pathogenic β-sheet-rich conformation (PrP^Sc^) capable of templating the misfolding of additional PrP^C^ molecules [1]. This self-propagating mechanism has profoundly influenced how researchers conceptualize other protein misfolding disorders, including Alzheimer’s disease, Parkinson’s disease, and bovine spongiform encephalopathy, which all exhibit common features such as aberrant protein conformers and progressive neuronal degeneration [3,4]. Despite these advances, critical gaps remain in understanding how prion-like mechanisms intersect with inflammatory processes—especially those involving bacterial components—during the onset and progression of neurodegeneration.

Emerging evidence suggests that bacterial lipopolysaccharide (LPS), a major outer membrane component of Gram-negative bacteria, may contribute to or exacerbate prion-like processes. In vitro experiments have shown that LPS can induce the formation of protease-resistant recombinant prion protein (moPrP^Res^) [5,6], suggesting intriguing possibilities about the role of bacterial factors in modulating pathological protein folding in vivo. Furthermore, chronic peripheral LPS exposure has been shown to aggravate the pathophysiology of other neurodegenerative proteinopathies, including Alzheimer’s and Parkinson’s diseases [7,8,9,10,11]. In these models, LPS activates the innate immune system [12,13], leading to microglial overactivation and persistent neuroinflammation within the central nervous system (CNS), key processes that accelerate neuronal damage [14,15,16]. Indeed, sustained peripheral LPS exposure can impair blood–brain barrier integrity, disrupt cytokine networks, and promote protein misfolding, thereby driving or amplifying neurodegenerative pathology [15,16,17,18,19,20].

Traditionally, experimental scrapie models employ intracerebral inoculation due to its relatively short incubation period. However, to better reflect natural prion transmission, peripheral routes such as subcutaneous injection have also been utilized, although they require longer incubation times [12,21]. Notably, subcutaneous administration has proven comparably effective in transmitting disease in various animal models and offers a more physiologically relevant approximation of how prions might disseminate from peripheral sites to the CNS [12,21].

Whether LPS alone—or in conjunction with a prion-like substrate—can trigger or hasten prion-like neurodegeneration in healthy animals remains uncertain. Addressing this question is vital for determining whether misfolded prion protein isoforms require inflammatory co-factors such as LPS to manifest full pathogenic potential in vivo, or whether they are intrinsically capable of initiating neurodegeneration. A clearer understanding of this interplay is especially relevant given the increasing recognition that many neurodegenerative disorders likely exist along a continuum of protein misfolding and inflammatory processes, rather than as wholly distinct disease entities.

In this study, we aimed to determine whether LPS-converted recombinant prion protein (moPrP^Res^), generated entirely in vitro by incubation with *Escherichia coli* O111:B4 LPS without traditional amplification methods like seeding or serial protein misfolding cyclic amplification (sPMCA), could independently induce prion-like neurodegeneration in wild-type mice following subcutaneous administration. In addition, we assessed whether chronic peripheral exposure to bacterial LPS alone—without classical prion agents—might trigger spongiform brain pathology and neurodegenerative changes. Finally, we explored the synergistic impact of co-administering LPS with a classical scrapie strain (Rocky Mountain Laboratory strain, RML) and moPrP^Res^ on disease progression, neuropathological severity, and prion protein deposition. By investigating these interactions, our study seeks to demonstrate whether bacterial inflammatory stimuli may contribute to early-stage prion and other protein-misfolding neurodegenerative disorders.

## 2. Results

### 2.1. Chronic LPS Exposure Increases Body Weight While RML and PrP^Res^ Treatments Suppress Growth in Mice

**Figure 1:**
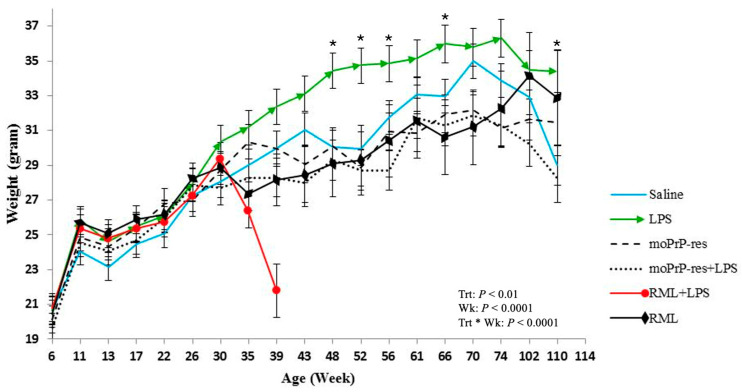
Monthly body weight measurements (mean ± SEM) of female FVB/N mice in six treatment groups—saline (negative control), bacterial lipopolysaccharide (LPS), mouse recombinant proteinase K-resistant prion protein (moPrP^Res^), moPrP^Res^ + LPS, Rocky Mountain Laboratory strain (RML) (positive control), and RML + LPS—monitored from 6 weeks of age until study endpoint. LPS or saline was continuously administered for six weeks via ALZET^®^ osmotic mini-pumps (ALZET, Cupertino, CA, USA), while a single subcutaneous injection of either moPrP^Res^ or RML was given at the time of pump implantation. Statistically significant differences in body weight were observed at multiple time points: LPS-treated mice weighed more than RML-treated mice at 35, 39, 43, 48, 52, and 66 weeks; more than saline controls at 48, 52, 56, 66, and 110 weeks (marked with an asterisk); more than RML + LPS mice at 35 and 39 weeks; more than moPrP^Res^ mice at 43, 48, 52, 56, 61, 66, 70, and 74 weeks; and more than moPrP^Res^ + LPS mice at 39, 43, 48, 52, 56, 61, 66, 70, 74, 102, and 110 weeks. Additional significant comparisons included moPrP^Res^ vs. RML (35 weeks), moPrP^Res^ vs. RML + LPS (35 and 39 weeks), moPrP^Res^ + LPS vs. RML + LPS (39 weeks), moPrP^Res^ + LPS vs. saline (56 and 70 weeks), RML vs. RML + LPS (39 weeks), and RML + LPS vs. saline (39 weeks).

Body weight was recorded monthly across all experimental groups starting at 6 weeks of age and continuing through to the endpoint of each cohort (74, 102, or 110 weeks). Treatment-specific differences in body weight emerged over time (Figure 1).

Mice treated with LPS exhibited a sustained increase in body weight, which became apparent around 30 weeks of age. Statistically significant differences (*p* < 0.05) in body weight were recorded at multiple time points when comparing LPS-treated animals to other groups: LPS versus RML at 35, 39, 43, 48, 52, and 66 weeks; LPS versus saline at 48, 52, 56, 66, and 110 weeks; LPS versus RML + LPS at 35 and 39 weeks; LPS versus moPrP^Res^ at 43, 48, 52, 56, 61, 66, 70, and 74 weeks; and LPS versus moPrP^Res^ + LPS at 39, 43, 48, 52, 56, 61, 66, 70, 74, 102, and 110 weeks.

The RML + LPS group showed a marked decline in body weight beginning around 30 weeks, with the lowest values observed between 35 and 39 weeks. This coincided with the earliest recorded clinical decline and mortality in this group.

moPrP^Res^-treated mice had higher mean body weights than the RML and RML + LPS groups at 35 and 39 weeks but remained consistently lower than LPS-treated mice from 43 to 74 weeks (*p* < 0.05). RML and moPrP^Res^ groups showed reduced body weights relative to saline controls beginning at 30 weeks and continuing to study end.

These data document treatment-related differences in body weight profiles across the lifespan of mice subjected to various prion and inflammatory stimuli.

### 2.2. Survival Outcomes and Clinical Progression in Mice Treated with LPS, moPrP^Res^, and RML

Five mice from each treatment group were euthanized at 11 weeks post-infection (wpi) for early pathological assessment. At this stage, all animals were clinically normal, with no visible behavioral or neurological abnormalities. The remaining ten mice per group were monitored for survival and development of clinical signs.

Clinical signs observed across groups included kyphosis, ataxia, tremors, head tilt, tail rigidity, bradykinesia, proprioceptive deficits, stupor, and sustained weight loss (>72 h). These criteria were used to determine humane endpoints.

**Figure 2:**
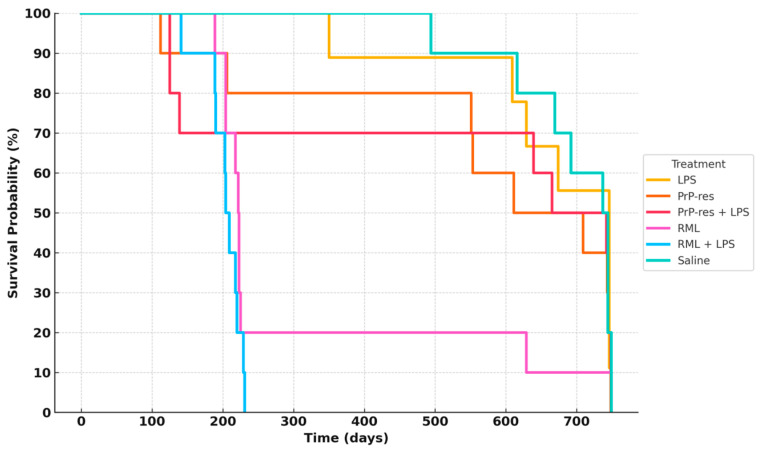
Survival analysis of terminally sick FVB/N female mice. All mice were monitored for clinical signs of prion disease until 750 days post-inoculation and were subsequently euthanized or found dead after maintaining clinical signs for a minimum of 72 h. Negative controls (saline-treated animals) exhibited no clinical signs of prion disease, with all being found dead in the cage without prior symptoms. Most treated animals were euthanized or found dead with clinical signs resembling prion disease. Twenty percent of the RML positive controls survived until the termination of the experiment. The combination of RML with LPS caused earlier mortality (within 100–150 days), with all mice dead by 200 days. Sixty percent of the moPrP^res^-treated mice died before termination, with four showing clinical signs of neurodegeneration. The moPrP^res^ + LPS treatment group had a mortality rate of 50%, with three mice showing clinical signs. Additionally, 40% of the LPS-treated animals exhibited clinical signs and were subsequently euthanized.

Figure 2 summarizes survival trajectories across all six groups.

In the RML-infected group, 80% of mice died by 200 days post-inoculation (dpi), while 10% survived up to approximately 700 dpi. In the RML + LPS group, 30% mortality occurred by 100 dpi, with complete mortality (100%) by 200 dpi. LPS-treated mice exhibited delayed mortality, with 10% death by 350 dpi and 40% cumulative mortality by 650 dpi; the remaining 60% survived to study termination at 110 weeks.

The moPrP^Res^ group exhibited a 20% mortality rate by 200 dpi, increasing to 60% by study end (750 dpi). The moPrP^Res^ + LPS group reached 30% mortality by 200 dpi and stabilized at 50% survival at endpoint.

### 2.3. Histopathological and Immunohistochemical Profiling of Neurodegeneration Across Treatment Groups

To complement the histopathological evaluation of neurodegeneration, all mice were monitored daily for clinical and behavioral abnormalities indicative of central nervous system dysfunction. Behavioral signs included kyphosis, ataxia, dysmetria, tremors, head tilt, tail rigidity, circling behavior, bradykinesia, proprioceptive deficits, stupor, loss of deep pain sensation, marked body weight loss, and diminished grooming—consistent with progressive neurodegeneration. Additional observations included reduced locomotor activity, hunched posture, impaired balance, and abnormal gait, all of which were particularly pronounced in terminally sick animals from the RML, RML + LPS, and moPrPRes + LPS groups. These behavioral abnormalities correlated closely with the highest vacuole counts and most extensive astrogliosis observed histologically. Although formal behavioral scoring was not performed, video documentation was collected to confirm the presence of consistent disease-associated phenotypes. These behavioral changes serve as functional correlates to the neuropathological findings and further support the conclusion that prion- and LPS-related treatments induced clinically relevant neurodegeneration in this experimental model.

**Figure 3:**
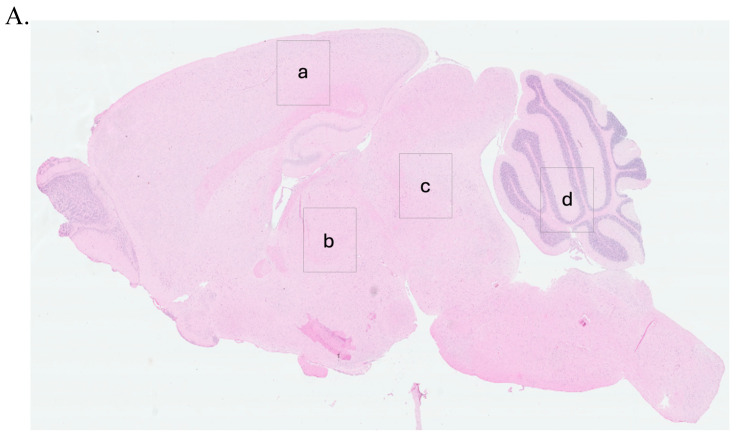

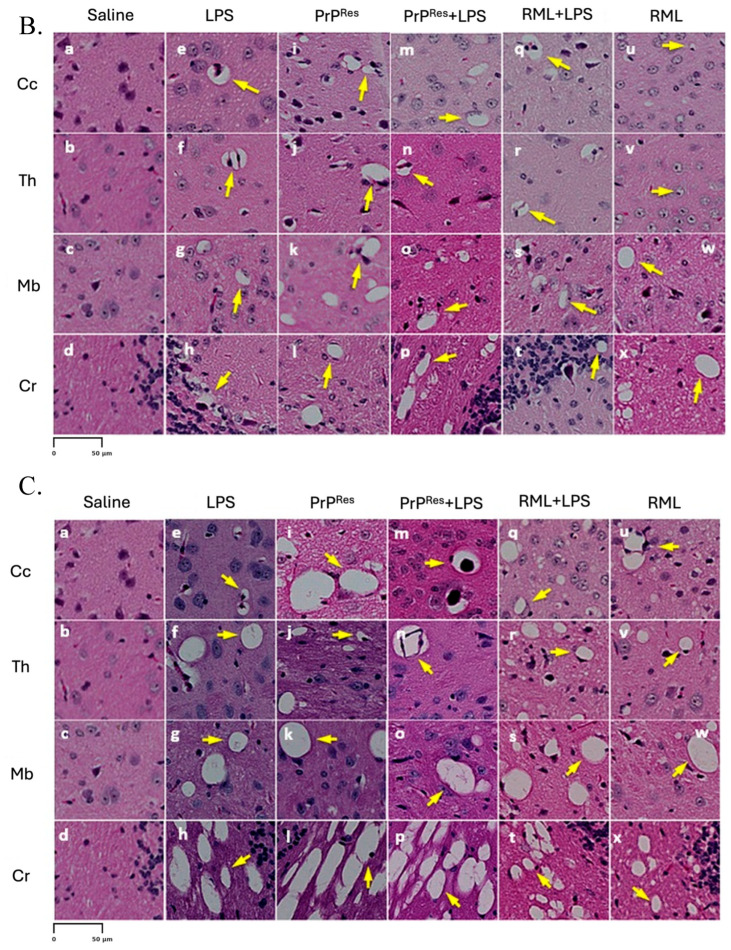
Prion-like vacuolation in hematoxylin and eosin (H&E) stained brain sections of FVB/N wild-type female mice at early (11 weeks postinfection) and terminal stages. (**A**) shows the anatomical representation of the brain indicating the primary regions analyzed: (a) Cerebral Cortex (Cc), (b) Thalamus (Th), (c) Midbrain (Mb), and (d) Cerebellum (Cr). These regions correspond to panels (a–x) presented in (**B**,**C**). (**B**) presents representative H&E-stained brain sections from mice at early stages (11 weeks post infection), treated subcutaneously for 6 weeks with saline (a–d), LPS (Escherichia coli O111:B4) (e–h), moPrP^Res^ (i–l), moPrP^Res^ + LPS (m–p), RML + LPS (q–t), or RML alone (u–x). (**C**) illustrates H&E-stained brain sections from terminally sick mice subjected to the same treatments and analyzed across the same regions: Cc (a, e, i, m, q, u), Th (b, f, j, n, r, v), Mb (c, g, k, o, s, w), and Cr (d, h, l, p, t, x). Yellow arrows highlight vacuolation in the brain parenchyma. Panels in both (**B**,**C**) are organized by treatment group to allow direct comparison of vacuolation patterns and severity at early and terminal stages. Vacuolation was more extensive in moPrP^Res^- and LPS-treated groups, particularly in terminal disease (**C**). (**A**) provides anatomical reference for both histological panels. Quantitative data and statistical analyses are provided in Section 2.4. Scale bar = 50 μm.

**Figure 4:**
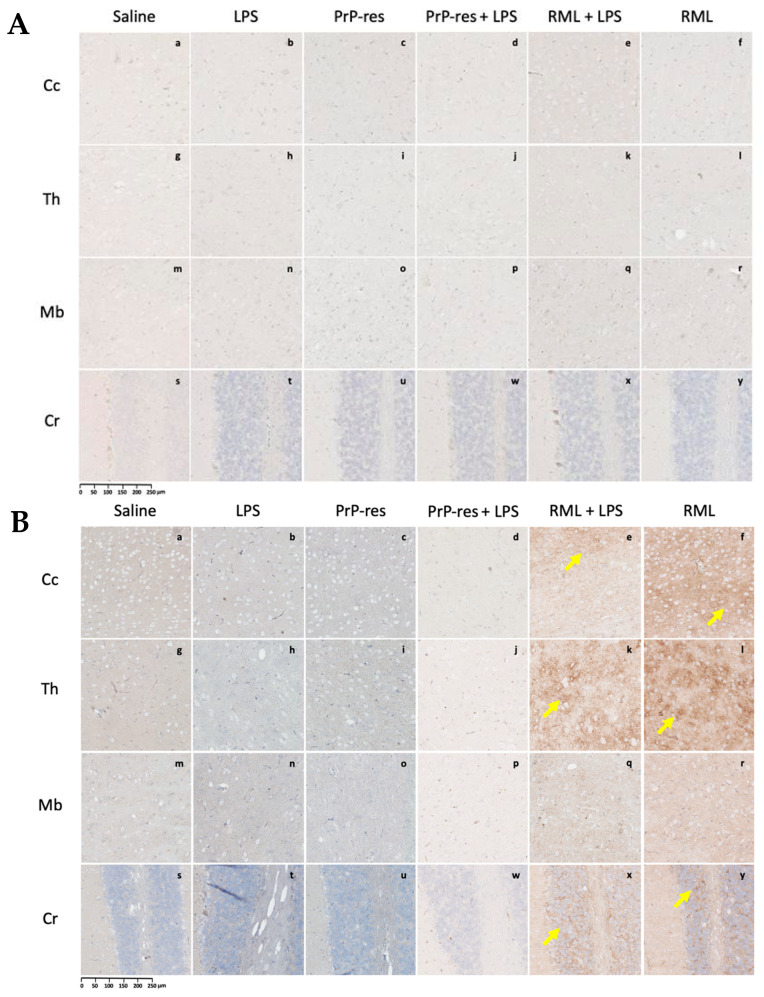
PrP^Sc^ deposition in immunohistochemically stained sections of the brains of FVB/N wild-type female mice. (**A**) Representative brain sections collected at 11 weeks post-inoculation from mice treated subcutaneously for 6 weeks with saline (a–d), LPS (Escherichia coli O111:B4) (e–h), moPrP^Res^ (i–l), moPrP^Res^ + LPS (m–p), RML + LPS (q–t), or RML alone (u–x). (**B**) Representative brain sections from terminally sick mice treated under the same conditions. Brain regions analyzed include the cerebral cortex (Cc; a, e, i, m, q, u), thalamus (Th; b, f, j, n, r, v), midbrain (Mb; c, g, k, o, s, w), and cerebellum (Cr; d, h, l, p, t, x). The anatomical locations of these regions are illustrated in Figure 3A for reference. PrP^Sc^ deposition was observed in the RML + LPS (q–t) and RML-alone (u–x) groups in both early (**A**) and terminal (**B**) stages, whereas no detectable deposition was found in the saline, LPS, moPrP^Res^, or moPrP^Res^ + LPS groups (a–p). Yellow arrows point to regions of PrP^Sc^ accumulation. Quantitative data and statistical comparisons are provided in Section 2.4. Scale bar represents 250 μm.

**Figure 5:**
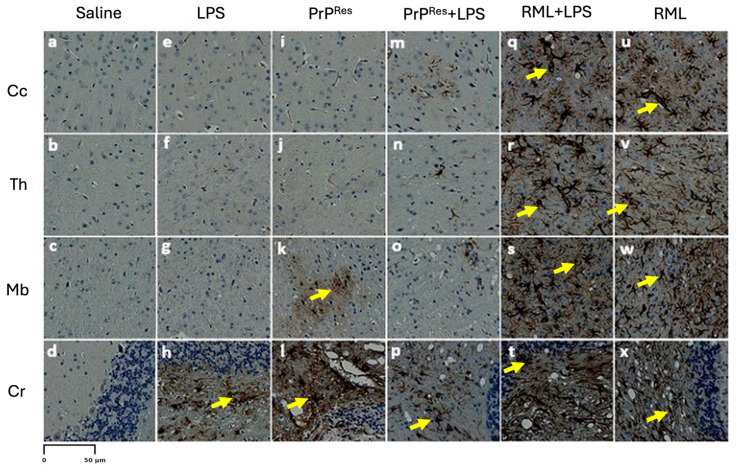
Astrogliosis in immunohistochemically stained sections of the brains of terminally sick FVB/N wild-type female mice. Representative stained brain sections from mice treated subcutaneously for 6 weeks with saline (a–d), LPS (*Escherichia coli* O111:B4) (e–h), moPrP^Res^ (i–l), moPrP^Res^ + LPS (m–p), RML + LPS (q–t), or RML alone (u–x). Brain regions analyzed include the cerebral cortex (Cc; a, e, i, m, q, u), thalamus (Th; b, f, j, n, r, v), midbrain (Mb; c, g, k, o, s, w), and cerebellum (Cr; d, h, l, p, t, x). The anatomical locations of these brain regions are illustrated in Figure 3A for reference. Astrogliosis was most prominent in the RML + LPS (q–t) and RML-alone (u–x) groups, while the saline, LPS, moPr^Res^, and moPrP^Res^ + LPS groups (a–p) exhibited minimal to moderate astrogliosis, primarily in the cerebellum. Yellow arrows point to astrocytes. Quantitative data and statistical comparisons are provided in Section 2.4. Scale bar represents 50 μm.

**Figure 6:**
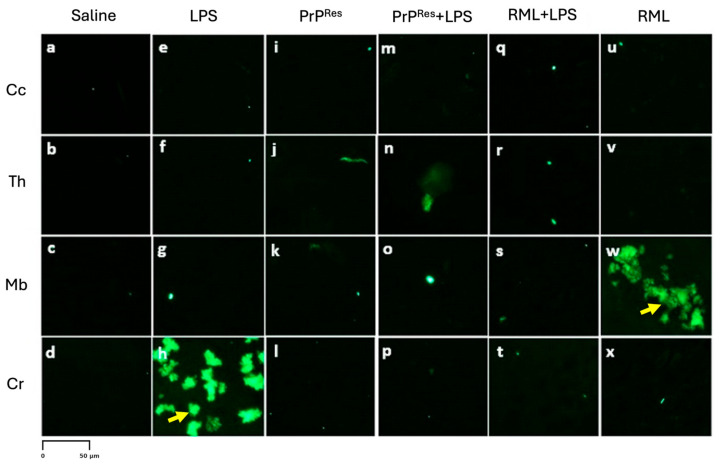
Amyloid plaque (Aβ) deposition in fluorescence-stained sections of the brains of terminally sick FVB/N wild-type female mice (10× magnification). Representative fluorescence-stained brain sections from mice treated subcutaneously for 6 weeks with saline (a–d), LPS (*Escherichia coli* O111:B4) (e–h), moPrP^Res^ (i–l), moPrP^Res^ + LPS (m–p), RML + LPS (q–t), or RML alone (u–x). Brain regions analyzed include the cerebral cortex (Cc; a, e, i, m, q, u), thalamus (Th; b, f, j, n, r, v), midbrain (Mb; c, g, k, o, s, w), and cerebellum (Cr; d, h, l, p, t, x). The anatomical locations of these brain regions are illustrated in Figure 3A for reference. Aβ deposition was predominantly observed in the midbrain (w) of RML-alone treated mice, while LPS-treated mice exhibited Aβ accumulation selectively in the cerebellum (h). No Aβ deposition was detected in the saline, moPrP^Res^, or moPrP^Res^ + LPS groups (a–p). Yellow arrows indicate Aβ deposition. Quantitative data and statistical comparisons are provided in Section 2.4. Scale bar represents 50 μm.

**Figure 7:**
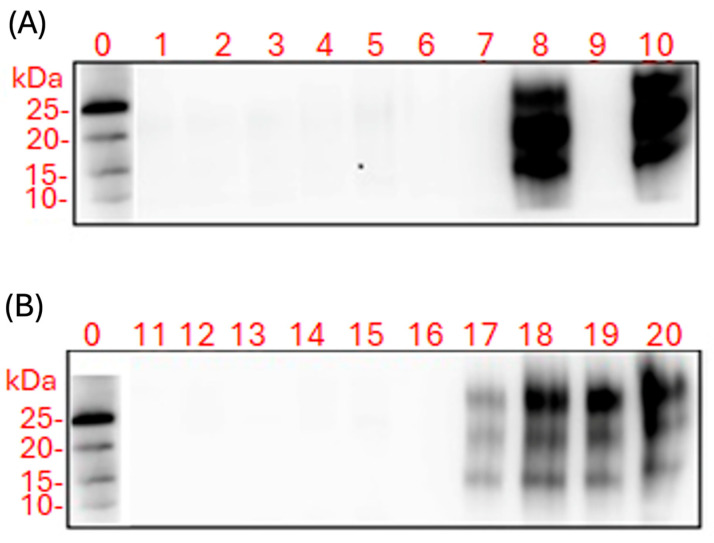
Western blot analysis of proteinase K (PK)-resistant prion protein (PrPSc) in brain (**A**) and spleen (**B**) homogenates from FVB/N female mice across treatment groups. All samples were digested with 50 µg/mL PK prior to electrophoresis to specifically detect protease-resistant PrPSc isoforms. Lane 0 shows the molecular weight marker (kDa), which was run on the same gel as all experimental samples. Lanes 1 and 11: saline controls; lanes 2 and 12: LPS-treated mice; lanes 3–4 and 13–14: moPrPRes-treated mice (11 wpi and terminal stage); lanes 5–6 and 15–16: moPrPRes + LPS-treated mice (11 wpi and terminal stage); lanes 7–8 and 17–18: RML + LPS-treated mice (11 wpi and terminal stage); lanes 9–10 and 19–20: RML-only group (11 wpi and terminal stage). PK-resistant PrPSc bands were detected only in the RML and RML + LPS groups, with more intense signals observed at the terminal stage. No PrPSc was detected in saline, LPS, moPrPRes, or moPrPRes + LPS groups. Non-digested (no-PK) controls were not included, as the aim was to assess only protease-resistant PrP. Housekeeping proteins (e.g., GAPDH) were not used because PK digestion degrades most cellular proteins. Equal protein loading was ensured by pre-quantification prior to electrophoresis. Negative control wells, including those seeded with saline brain homogenates and untreated L929 cells, showed no detectable PrP^Sc^ signal. Similarly, no PrP^Sc^ was detected in wells treated with brain homogenates from LPS-, moPrP^Res^-, or moPrP^Res^ + LPS-treated animals. All signal levels in these groups remained below the assay’s detection threshold (Figure 8).

**Figure 8:**
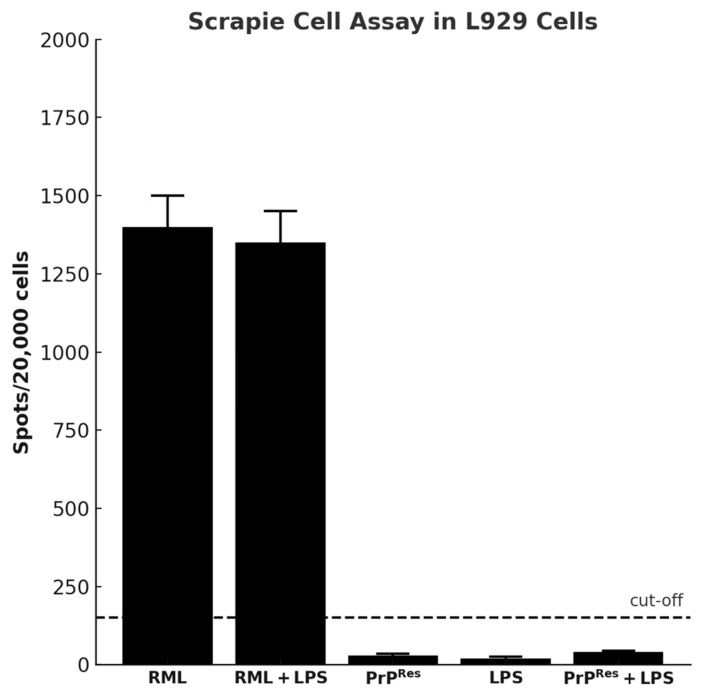
Detection of prion infectivity using the scrapie cell assay (SCA) in L929 mouse fibroblast cells exposed to brain homogenates from terminally sick FVB/N female mice (mean ± SEM). Brain homogenates from three terminally sick animals per treatment group (RML, RML + LPS, moPrP^Res^, moPrP^Res^ + LPS, and LPS alone) were applied to L929 cells in a serial dilution range of 0.1% to 0.0001%, with six technical replicates per dilution. The number of PrP^Sc^-positive foci (spots) was quantified per 20,000 cells. Only homogenates from RML and RML + LPS groups produced values above the detection threshold, indicating infectivity. The horizontal dashed line represents the assay cut-off (150 spots/20,000 cells). No significant infectivity was detected in cells exposed to homogenates from LPS, PrP^Res^, or PrP^Res^ + LPS groups.

To guide the interpretation of region-specific pathology, Figure 3A provides a schematic illustration of the four major brain regions analyzed in this study: cerebral cortex (Cc)**,** thalamus (Th)**,** midbrain (Mb)**,** and cerebellum (Cr). These regions were selected based on their relevance to prion-related and inflammation-induced neurodegeneration. This anatomical reference supports the localization of vacuolar changes and immunohistochemical signals described in subsequent subfigures (Figure 3B, panels a–x) and throughout Figure 4, Figure 5, Figure 6, Figure 7 and Figure 8.

Histological and immunohistochemical assessments were conducted to characterize neuropathological changes across all experimental groups, including saline controls, LPS-treated, moPrP^Res^-, RML-, and co-treated mice. Analyses focused on four principal pathological features: (1) spongiform vacuolation, (2) PrP^Sc^ accumulation, (3) astrogliosis as measured by GFAP immunoreactivity, and (4) amyloid-beta (Aβ) plaque deposition.

Representative images from H&E staining, PrP^Sc^ immunohistochemistry, GFAP staining, and Aβ plaque detection are provided in Figure 3, Figure 4, Figure 5 and Figure 6. Quantitative assessment of vacuole counts across four brain regions—Cc, Th, Mb, and Cr—was performed at three key time points: 11 wpi (euthanization stage), terminal disease stage (sick animals), and study endpoint (termination stage).

Data reveal group- and region-specific differences in the severity and anatomical distribution of neuropathology, supporting further comparisons across disease models induced by prion infection, recombinant PrP^Res^ administration, and chronic systemic LPS exposure.

#### 2.3.1. LPS-Induced Cerebellar Degeneration Without PrP^Sc^ Accumulation

Histopathological evaluation of brain sections from LPS-treated mice revealed progressive vacuolar degeneration, particularly affecting the Cr. At 11 wpi, euthanized animals in this group showed mild spongiform changes in all four examined brain regions—Cc, Th, Mb, and Cr (Figure 3B, panels e–h). Vacuole counts at this stage were modest and comparable to those observed in saline-treated controls (Further details are provided in Section 2.4).

In contrast, terminally ill LPS-treated mice exhibited increased vacuolation, with the most prominent changes observed in the Cr and Mb (Figure 3C, panels e–h). Quantitative vacuole analysis confirmed elevated counts in these regions relative to both the 11 wpi LPS group and the saline controls, while overall vacuole numbers remained lower than those recorded in RML-infected mice.

Immunohistochemical staining for PrP^Sc^ in LPS-treated animals showed no detectable signals at either 11 wpi or terminal disease stages (Figure 5 and Figure 6, panels e–h). GFAP immunostaining indicated localized astrogliosis confined to the cerebellum in terminal LPS-treated mice (Figure 5, panel h), with minimal astrocytic activation observed in the Cc, Th, or Mb regions (Figure 5, panels e–g).

Amyloid-beta (Aβ) immunostaining revealed plaque deposition exclusively in the cerebellum of terminally ill LPS-treated animals (Figure 8, panel h). No Aβ-positive staining was detected in other brain regions or in any saline, RML-, or moPrP^Res^-treated mice (Figure 6, panels d, l, p, x).

#### 2.3.2. Recombinant moPrP^Res^ Induces Vacuolar Neurodegeneration Without PrP^Sc^ or Amyloid Plaque Deposition

Histological analysis of moPrP^Res^-treated mice euthanized at 11 wpi revealed low-grade spongiform vacuolation in the Cc, Th, Mb, and Cr (Figure 3B, panels i–l). No vacuoles were observed in the saline-treated control animals (Figure 3B, panels a–d). Quantitative vacuole counts (see Section 2.4 for more details) confirmed modest elevations across all regions in the moPrP^Res^ group, with the Cr exhibiting the highest susceptibility.

Terminally sick moPrP^Res^-treated mice displayed substantially increased vacuole formation in all four brain regions, as shown in H&E-stained sections (Figure 3C, panels i–l). The spatial distribution of vacuoles was similar to that observed in RML-infected animals; however, total vacuole counts remained lower, and vacuoles appeared larger in some regions.

Immunohistochemistry for disease-associated prion protein (PrP^Sc^) yielded no detectable signal in moPrP^Res^-treated mice at either 11 wpi or terminal stages (Figure 6, panels i–l), consistent with the absence of signal in saline-treated controls (Figure 6, panels a–d). GFAP immunostaining revealed mild to moderate astrocytic activation, particularly in the Cr (Figure 5, panel l) and Mb (Figure 5, panel k), with minimal glial reactivity in the Cc and Th (Figure 5, panels i–j).

Amyloid-β immunostaining was negative in all examined brain regions of moPrP^Res^-treated and moPrP^Res^ + LPS mice (Figure 6, panels i–l and m-p, respectively), indicating no evidence of amyloid plaque deposition. In contrast, LPS-treated mice displayed Aβ accumulation exclusively in the Cr (Figure 6, panel h), while RML-treated mice showed Aβ accumulation restricted to the Mb (Figure 6, panel w) in late-stage disease. Notably, the RML + LPS co-treated group did not exhibit detectable Aβ staining in any brain region (Figure 6, panels q–t).

#### 2.3.3. Combined moPrP^Res^ and LPS Treatment Induces Neurodegeneration Without PrP^Sc^ Accumulation and Minimal Amyloid Deposition

Mice receiving combined moPrP^Res^ + LPS treatment exhibited no clinical signs of disease at 11 wpi. Histopathological evaluation revealed mild vacuolation in the Cc, Th, Mb, and Cr, comparable to those observed in the LPS-only and moPrP^Res^-only groups and absent in saline controls (Figure 3B, panels m–p). Immunohistochemical staining for PrP^Sc^ showed no detectable deposition in any brain region at this stage (Figure 4A, panels m–p), mirroring the PrP^Sc^-negative profiles of the saline, LPS, and moPrP^Res^ groups.

At terminal stages, moPrP^Res^ + LPS-treated animals developed prominent vacuolation across all four brain regions (Figure 3C, panels m–p). Quantitative vacuole analysis revealed that although the total vacuole counts were lower than in RML-inoculated animals, the vacuoles were consistently larger in size. This pattern replicated the morphological features seen in the moPrP^Res^-only group and suggests that systemic LPS exposure augmented the neurodegenerative phenotype without increasing classical lesion burden.

Immunostaining for PrP^Sc^ remained negative in all terminal moPrP^Res^ + LPS-treated mice (Figure 6, panels m–p), confirming the absence of prion propagation and reinforcing the non-infectious nature of moPrP^Res^, even under pro-inflammatory conditions. Moderate astrogliosis was evident in the Cr and Th, with increased GFAP immunoreactivity compared to controls (Figure 5, panels m–p), but the intensity remained less than that observed in RML and RML + LPS-treated mice.

Amyloid-β staining revealed sparse plaque deposition limited to the Th and Mb (Figure 8, panels n and o), with no Aβ detected in the Cc or Cr (Figure 8, panels m, o, p). These results indicate that while chronic inflammation may facilitate limited Aβ accumulation, co-treatment with moPrP^Res^ does not markedly enhance amyloidogenic processes.

Collectively, these data demonstrate that the combined administration of moPrP^Res^ and LPS induces a widespread but atypical form of neurodegeneration characterized by large vacuoles, moderate gliosis, and minimal amyloid deposition, occurring in the absence of PrP^Sc^ accumulation.

#### 2.3.4. LPS Enhances PrP^Sc^ Accumulation and Neurodegeneration in RML-Infected Mice Without Inducing Amyloid Plaques

At 11 wpi, mice co-treated with RML and LPS, though still asymptomatic, showed early neurodegenerative changes characterized by mild vacuolation in the Cc, Th, Mb, and Cr (Figure 3B, panels q–t). Immunohistochemical analysis revealed more extensive PrP^Sc^ deposition in these regions compared to mice treated with RML alone at the same time point (Figure 4A, panels q–t vs. u–x), suggesting that systemic LPS exposure accelerates prion propagation during the preclinical phase.

In terminally ill RML + LPS mice, vacuolation was widespread and severe across all examined brain regions, with the Cr and Th most prominently affected (Figure 3C, panels q–t). While the overall lesion severity visually matched or exceeded that in the RML-only group, quantitative analysis indicated a slightly lower total vacuole count in the co-treated animals. Nonetheless, PrP^Sc^ deposition was markedly elevated in the Cc, Th, and Cr of RML + LPS mice at terminal stages (Figure 4B, panels q–t), reinforcing the role of systemic inflammation in enhancing prion replication and neuroinvasion.

Astrogliosis was similarly intensified, as evidenced by strong GFAP immunoreactivity throughout all brain regions (Figure 5, panels q–t). The astrocytic response in the RML + LPS group was notably more robust than in the RML-only group (Figure 5, panels u–x), indicating that co-stimulation with LPS amplifies neuroinflammatory processes associated with prion disease.

Despite the advanced neurodegeneration and extensive PrP^Sc^ pathology, no amyloid-β (Aβ) plaques were detected in any brain region of the RML + LPS group (Figure 6, panels q–t), in contrast to the midbrain-specific Aβ accumulation observed in RML-only treated animals (Figure 6, panels u–x). These results indicate that although systemic LPS exposure intensifies prion-associated pathology, it does not promote Aβ plaque formation under these experimental conditions.

#### 2.3.5. LPS Enhances RML-Induced Neurodegeneration and PrP^Sc^ Accumulation Without Promoting Amyloid-β Deposition

At 11 wpi, hematoxylin and eosin (H&E) staining revealed minimal vacuolation in most brain regions in both the RML-only and RML + LPS treatment groups, consistent with the preclinical stage. Similar vacuolation patterns were observed in moPrP^Res^ and moPrP^Res^ + LPS-treated mice (Figure 3B, i–p), although all these groups exhibited more vacuolation than the negative saline controls (Figure 3B, a–d).

In terminally sick animals, H&E staining showed widespread vacuolation in the Cc, Th, Mb, and Cr in the RML + LPS-treated group (Figure 3C, q–t). In contrast, the RML-only group showed less extensive and less intense vacuolation in the same brain regions (Figure 3C, u–x).

Immunohistochemical analysis showed higher levels of PrP^Sc^ deposition in the RML + LPS-treated group, particularly in the Cc, Th, and Cr, compared to the RML-only group (Figure 4B, q–x). While the overall spatial distribution of PrP^Sc^ deposition was similar between RML and RML + LPS groups, the staining intensity appeared subjectively higher in the RML + LPS group, suggesting enhanced accumulation without major shifts in regional tropism. No PrP^Sc^ deposits were observed in saline, LPS, moPrP^Res^, or moPrP^Res^ + LPS-treated mice at either 11 wpi or at terminal stages (Figure 4B, a–p).

Glial fibrillary acidic protein (GFAP) immunostaining revealed strong astrogliosis across all analyzed brain regions in the RML + LPS-treated mice (Figure 5, q–t), while RML-only mice exhibited moderate GFAP staining (Figure 7, u–x). Mild to moderate astrogliosis, primarily localized to the Cr, was also observed in the LPS, moPrP^Res^, and moPrP^Res^ + LPS groups (Figure 5, e–p).

Amyloid-β (Aβ) staining identified plaques in the Mb and Cr of the RML + LPS and LPS-only treatment groups (Figure 8, panels h and t). No Aβ plaques were detected in brain sections from RML-only, moPrP^Res^, moPrP^Res^ + LPS, or saline-treated mice (Figure 6, panels a–g, i–p, u–x).

#### 2.3.6. PrP^Sc^ Detected Only in RML- and RML + LPS-Treated Mice: Absence in LPS and moPrP^Res^ Groups

Western blot analysis was performed on proteinase K (PK)-treated brain and spleen homogenates collected from FVB/N female mice at both 11 wpi and at terminal disease stages (Figure 7).

No PK-resistant PrP bands were detected in brain or spleen homogenates from saline-treated control animals (Figure 7, lanes 1 and 11). Similarly, mice treated with LPS, moPrP^Res^, or moPrP^Res^ + LPS showed no detectable PrP^Sc^ in either brain or spleen homogenates at any time point (Figure 7, lanes 2–7 and 12–16).

In RML-treated mice, PrP^Sc^ was not detected in brain homogenates at 11 wpi (Figure 7A, lane 9), but was present in spleen homogenates from the same time point (Figure 7B, lane 19). In terminally sick RML-treated mice, PK-resistant PrP was observed in both brain and spleen homogenates (Figure 7A, lane 9; Figure 7B, lane 20).

In the RML + LPS group, PrP^Sc^ was detectable in brain homogenates of terminally sick mice (Figure 7A, lane 10), as well as in spleen homogenates at both 11 wpi and terminal stages (Figure 7B, lanes 17 and 18).

#### 2.3.7. Cell-Based Assay Confirms Infectivity Only in RML and RML + LPS Brain Homogenates

A cell-based infectivity assay was performed using the L929 mouse fibroblast line exposed to brain homogenates from terminally sick mice across all treatment groups. Following culture on ELISPOT plates, cells were subjected to proteinase K (PK) digestion and immunodetection to assess the presence of PK-resistant PrP^Sc^.

In contrast, strong PrP^Sc^-positive signals were observed in cells treated with brain homogenates from RML-infected mice and from the RML + LPS co-treatment group (Figure 8). These results indicate the presence of infectious, PK-resistant prion protein in these groups.

### 2.4. Quantitative Analysis of Spongiform Vacuolation Across Brain Regions 

Spongiform vacuolation was quantified in four brain regions—the Cc, Th, Mb, and Cr—at three experimental stages: 11 wpi (Euthanization), terminal clinical illness (Sick), and study endpoint (Termination).

#### 2.4.1. Euthanization Stage (11 wpi)

At 11 wpi, vacuole counts were generally low across all treatment groups (n = 5), consistent with the absence of clinical disease. The RML-infected group exhibited the highest early-stage vacuolation, particularly in the Cc (11,853 ± 412, *p* < 0.0001), Mb (5871 ± 305, *p* < 0.0001), and Cr (5450 ± 846, *p* < 0.0001), all significantly elevated compared to saline controls (349.4 ± 2.1, 88.4 ± 2.1, and 139.6 ± 2.9, respectively). These data suggest that RML induces early subclinical neuropathology prior to the onset of clinical symptoms.

The LPS-only group showed mild vacuolation, notably in the cerebellum (1152 ± 120, *p* = 0.002) and midbrain (287 ± 42, *p* = 0.025), while vacuole counts in the cerebral cortex (233 ± 76, *p* = 0.128) and thalamus (212 ± 50, *p* = 0.048) were not consistently elevated or statistically significant relative to saline.

Mice treated with moPrP^Res^ and moPrP^Res^ + LPS exhibited uniformly low vacuole counts across all regions (typically 45–150 vacuoles), with no significant differences from saline controls, indicating minimal neurodegeneration at this stage.

Strikingly, the RML + LPS group displayed markedly reduced vacuolation compared to the RML-only group across all regions examined: cortex (60 ± 1, *p* < 0.0001), midbrain (48 ± 2, *p* < 0.0001), cerebellum (54 ± 3, *p* < 0.0001), and thalamus (37 ± 1, *p* < 0.0001). These findings suggest that co-administration of LPS may suppress or delay the onset of prion-induced vacuolar pathology during the early, asymptomatic phase of disease progression.

#### 2.4.2. Termination Stage (110 wpi)

At the end of the experiment (110 wpi), among animals that remained clinically asymptomatic, vacuole counts across all groups were low and showed no substantial variation. The saline group (n = 4) exhibited consistent vacuole intensities across brain regions, with mean ± SEM values as follows: Cr (197.6 ± 1.7), Cc (209.4 ± 1.8), midbrain (209.0 ± 3.1), and Th (208.4 ± 2.9).

In contrast, most treatment groups at this stage had only two surviving animal per treatment, precluding reliable statistical comparisons. The LPS-treated mouse (n = 2) exhibited vacuole intensities of 200.9 (Cr), 217.2 (Cc), 215.6 (Mb), and 215.1 (Th), slightly elevated compared to saline means but not substantially different. Similarly, the moPrP^Res^ + LPS group (n = 2) showed values close to those of the saline group—for example, 195.2 in the cerebellum and 209.2 in the cortex—suggesting no clear trend toward increased or decreased vacuolation.

The RML group (n = 2) also displayed vacuole intensities similar to saline in all assessed regions, such as 202.6 in the Cr and 209.5 in the cortex, indicating an absence of significant spongiform change in animals that survived to this time point. Notably, all RML + LPS-treated animals succumbed at 200 dpi, and thus data for this group are not available for this stage.

In summary, vacuolation remained minimal in all groups surviving to the termination stage, and the small number of animals in most treated groups limited the statistical power of comparisons. These observations support the interpretation that substantial spongiform pathology had not developed in clinically asymptomatic animals at this time point.

#### 2.4.3. Sick Animals (Terminal Clinical Stage)

In terminally sick animals, all prion- and LPS-related treatment groups exhibited extensive vacuolar pathology across multiple brain regions. RML-infected mice (n = 3) showed the highest vacuole counts overall, with particularly elevated values in the Cr (1623.7 ± 305.5) and Th (1491.3 ± 345.9), consistent with advanced neurodegeneration.

However, the RML + LPS-treated group (n = 4) exhibited similarly high vacuolation, including 1603.0 ± 258.5 in the Cc and 1902.0 ± 325.1 in the Th, in some cases exceeding the values observed in the RML-only group. These differences were not statistically significant (Cc: *p* = 0.10; Th: *p* = 0.15), suggesting that LPS co-treatment did not appreciably alter the severity of vacuolation in animals that progressed to clinical disease.

The moPrP^Res^ + LPS group (n = 2) also demonstrated pronounced vacuolation across regions, with 1643.0 ± 382.0 in the cerebellum, 942.5 ± 113.5 in the cortex, and 841.0 ± 198.0 in the thalamus. Statistical comparisons revealed no significant differences in vacuole counts between moPrP^Res^ + LPS and RML groups in the Cc (*p* = 0.17) or Th (*p* = 0.44), nor between moPrP^Res^ + LPS and RML + LPS in the same regions (*p* = 0.63 and *p* = 0.43, respectively).

These results indicate that co-administration of LPS with either RML or moPrP^Res^ results in vacuolar pathology comparable to that of classical prion infection alone. At the terminal clinical stage, LPS neither exacerbated nor mitigated spongiform degeneration, suggesting that its modulatory effects are more relevant during earlier phases of disease progression.

Quantitative comparison of vacuolation severity between the moPrP^Res^-only group (n = 3) and the RML-infected group (n = 3) revealed region-specific differences. In the thalamus, vacuole counts were significantly higher in RML-treated animals (1089.0 ± 45.7) compared to moPrP^Res^-treated mice (908.8 ± 31.4, *p* = 0.037), indicating more pronounced neurodegeneration in this region. In contrast, vacuole counts in the cerebral cortex (Cc: 910.5 ± 40.0 vs. 813.8 ± 37.5, *p* = 0.15) and midbrain (Mb: 1002.0 ± 117.4 vs. 771.7 ± 35.1, *p* = 0.182) showed a trend toward higher values in the RML group, but these differences did not reach statistical significance. Notably, the cerebellum exhibited nearly identical vacuole burdens across both groups (Cr: 825.9 ± 37.0 vs. 829.4 ± 92.7, *p* = 0.97), suggesting a shared vulnerability to neurodegeneration in this region. These findings support the conclusion that moPrP^Res^ alone can induce substantial spongiform pathology, with severity overlapping that of classical prion infection in several brain regions.

#### 2.4.4. Comparative Summary and Statistical Outcomes

Comparative analysis of vacuole formation across all three stages—euthanization (11 wpi), termination (110 wpi), and clinical sickness—revealed a clear progression of neuropathology over time, with statistically robust differences becoming most apparent at the terminal stage. At 11 wpi, vacuole counts were significantly elevated in the RML group across all brain regions compared to other groups (*p* < 0.001), yet these changes remained subclinical, and sample sizes were sufficient (n = 5) for reliable statistical testing. Notably, co-treatment with LPS (RML + LPS and moPrP^Res^ + LPS) paradoxically suppressed vacuole accumulation below levels seen in the respective prion-only groups, suggesting an early-stage modulatory effect of systemic inflammation.

In contrast, at the termination stage (110 wpi), vacuole counts remained low across all groups, and most animals were clinically asymptomatic. However, this stage was limited by small sample sizes—particularly for the LPS (n = 1), moPrP^Res^ + LPS (n = 2), and RML (n = 2) groups—precluding definitive statistical conclusions despite descriptive trends. For example, the RML group displayed modestly higher vacuole intensities than saline controls, especially in the cerebellum and thalamus, but these differences were not statistically significant. The moPrP^Res^ + LPS group showed values nearly identical to saline mice, reinforcing the notion that combined LPS exposure may dampen prion-induced pathology under some conditions.

Statistically significant intergroup differences were observed only at the sick stage, where vacuolation was extensive and distinct. These findings emphasize that spongiform degeneration in this model is a time-dependent process, with early LPS-associated neuroinflammation detectable but not amplifying prion toxicity, and late-stage pathology requiring sufficient disease burden and group size to draw robust conclusions.

## 3. Discussion

### 3.1. Overview of Key Findings

This study makes three major contributions to our understanding of prion-like and inflammatory neurodegenerative processes. First, we show that a recombinant prion protein (moPrP^Res^) generated entirely in vitro by incubation briefly with LPS—without either seeding factors or serial protein misfolding cyclic amplification (sPMCA)—can provoke a distinctive neurodegenerative phenotype in wild-type FVB/N mice when administered subcutaneously. Second, we provide evidence that 6-week subcutaneous exposure to bacterial LPS, in the absence of a classical prion agent, is sufficient to induce Alzheimer’s-like spongiform brain pathology. Third, we demonstrate that the co-administration of LPS with the RML prion strain significantly accelerates disease onset, enhances neuropathological severity, and amplifies prion protein deposition, underscoring the powerful synergistic interplay between inflammatory triggers and classical prion agents.

Taken together, these findings highlight how bacterial factors and protein misfolding processes can converge or operate in parallel to drive neurodegenerative pathology. They also challenge the prevailing assumption that prion-related neurodegeneration is predicated solely on the accumulation of detectably protease-resistant prion protein (PrP^Sc^) [1,2]. Instead, our data suggest multiple molecular routes—both prion-dependent and prion-independent—can converge upon a final common pathway of neuronal damage.

### 3.2. Infectivity and Pathogenic Potential of In Vitro-Generated moPrP^Res^

A key outcome of this research is that moPrP^Res^, produced through in vitro conversion using *E. coli* 0111:B4 LPS [5,6], can induce a pronounced spongiform encephalopathy in FVB/N mice without overt PrP^Sc^ accumulation. This finding expands upon prior demonstrations that exogenous cofactors, such as phospholipids or RNA, can foster prion protein misfolding in vitro [3,4]. However, in many cases, the resulting protease-resistant prion forms fail to cause disease in vivo unless further amplified by serial PMCA or supplemented with seeding factors [3,4]. By contrast, the moPrP^Res^ described herein was generated without additional amplification steps, indicating that a purely recombinant misfolded prion protein can be pathogenic in a wild-type host.

The neuropathological profile—featuring spongiform changes, significant astrogliosis (especially in the cerebellum), and a collection of clinical signs—resembles a prion-like disorder but deviates from classical scrapie in that PrP^Sc^ was undetectable by Western blot, immunohistochemistry, or scrapie cell assays [22]. This discrepancy strongly suggests that misfolded or partially aggregated conformers of PrP may be neurotoxic, even in the absence of large, protease-resistant deposits [3,4]. Alternatively, moPrP^Res^ might invoke alternative neurodegenerative cascades, independent of the canonical prion replication pathway that yields abundant PrP^Sc^.

It should be noted that the biochemical features of the moPrP^Res^ used in this study were previously characterized in a previous publication [5], in which recombinant Syrian hamster PrP (90–232) incubated with LPS underwent a clear conformational shift from α-helical to β-sheet-rich structure, as shown by circular dichroism spectroscopy. Furthermore, SDS-PAGE analysis of the LPS-treated PrP revealed a protease K–resistant core of approximately 12 kDa, consistent with partial resistance characteristic of prion-like aggregates. These findings support the conclusion that the LPS-converted moPrP^Res^ adopts a stable, β-sheet-rich conformation, distinct from classical PrP^Sc^ but capable of inducing neurodegeneration. The current study builds on that structural evidence by demonstrating its pathological effects in vivo.

### 3.3. Lack of Detectable PrP^Sc^

The lack of classical protease-resistant prion aggregates in moPrP^Res^-treated mice raises fundamental questions about the centrality of PrP^Sc^ deposition to prion disease. It is widely accepted that the accumulation of PrP^Sc^ is the hallmark of prion disorders [1,2], yet increasing evidence indicates that smaller oligomeric or intermediate conformers may be the key mediators of neurotoxicity [3,4]. Our data suggest that moPrP^Res^ can elicit pathological changes and clinical signs in the absence of overt PrP^Sc^ accumulation. This aligns with theories in other neurodegenerative proteinopathies (e.g., Alzheimer’s disease) that smaller, soluble aggregates, rather than large fibrillar deposits, may drive neuronal dysfunction.

Importantly, the SAF83 monoclonal antibody used in our immunohistochemistry and ELISPOT assays recognizes an epitope within residues 145–155 of PrP and is validated for detecting classical PrP^Sc^ isoforms. However, it remains unverified whether SAF83 can bind recombinant moPrP^Res^ generated via LPS conversion. Thus, failure to detect prion aggregates with this antibody cannot definitively exclude the presence of alternative prion-like conformers. Notably, Thioflavin S staining revealed amyloid plaques predominantly in the LPS-only and RML-treated groups, whereas mice treated with moPrP^Res^ or RML + LPS exhibited minimal or near-undetectable amyloid deposition. These findings suggest that the aggregates formed in moPrP^Res^-treated mice are either structurally distinct from canonical amyloid fibrils or present at levels below detection threshold. The combined absence of protease-resistant PrP in Western blot, immunohistochemistry, and SCAs further supports the interpretation that structurally divergent, nonclassical species may be responsible for the observed neurotoxicity in the moPrP^Res^ group—consistent with emerging models of oligomer-mediated neurodegeneration.

### 3.4. Distinctions from RML-Derived Disease

Comparisons with RML (brain homogenate) treatments exposed substantial differences in incubation period, mortality rates, and neurodegeneration patterns. Whereas RML-inoculated mice typically succumbed by ~200–250 days post-infection (dpi), moPrP^Res^-treated animals exhibited significantly prolonged survival, with some remaining alive beyond 700 dpi. This disparity likely reflects the comparatively high infectivity of prion-containing brain homogenates [12] and the presence of pro-inflammatory or cofactor molecules that accelerate disease [4,12]. The relatively “pure” nature of moPrP^Res^, lacking these additional elements, may impede peripheral prion propagation and delay its ultimate neurotoxic effects. From a translational standpoint, these findings reinforce the notion that the composition of the inoculum—including inflammatory mediators and cofactors—critically influences disease onset and outcome [12,22].

In this study, a single subcutaneous injection of either moPrP^Res^ (amino acids 29–232) at a dosage of 45 µg per mouse or RML homogenate containing 10^7^ ID_50_ units of scrapie prions was administered at the time of osmotic pump implantation. The moPrP^Res^ protein was produced recombinantly and delivered in a defined volume of 200 µL per mouse. Unlike the RML inoculum, which contains infectious PrP^Sc^ along with a heterogeneous mixture of cellular debris, lipids, and inflammatory cofactors derived from the terminally ill brain, moPrP^Res^ represents a structurally misfolded but non-infectious form of the prion protein, devoid of seeding activity. As such, no direct equivalence can be drawn between the administered protein dose and the infectious titer of the RML group. The aim of using moPrP^Res^ was not to simulate infectivity, but to evaluate whether a misfolded, protease-resistant prion conformer could elicit neurotoxicity in vivo in the absence of replication. This distinction is underscored by the lack of detectable PrP^Sc^ in moPrP^Res^-treated animals, as assessed by Western blot, immunohistochemistry, and SCA [6,22]. While RML-treated mice exhibited clear evidence of infectivity and PrP^Sc^ accumulation, the pathological effects observed in the moPrP^Res^ group likely arise from alternative mechanisms such as oligomeric toxicity, immune activation, or prion-like signaling processes rather than classical strain propagation.

### 3.5. LPS-Induced Neurodegeneration

#### 3.5.1. Chronic LPS Infusion and Neuroinflammatory Damage

An interesting finding of this study is that a sustained six-week subcutaneous infusion of LPS alone was sufficient to induce extensive spongiform vacuolation, robust neuroinflammation, and approximately 40% mortality in wild-type FVB/N mice. While it is well established that acute or bolus injections of LPS can trigger transient neuroinflammation [8,9,15,16,23], the Alzheimer’s-like neuropathology observed here—including pronounced spongiosis and detectable Aβ accumulation—highlights the potent neurodegenerative potential of chronic, low-dose endotoxemia in otherwise healthy animals.

These observations are consistent with the concept of “metabolic endotoxemia,” in which persistent low-level translocation of bacterial endotoxins from the gut into systemic circulation initiates widespread immune activation. Peripheral LPS exposure has been shown to impair blood–brain barrier (BBB) integrity, prime microglia toward a pro-inflammatory phenotype, and upregulate key mediators such as TNF-α, IL-1β, MCP-1, and NF-κB p65, creating a chronic inflammatory environment that may persist for months [16,24,25]. This prolonged neuroinflammatory state can ultimately culminate in progressive neuronal injury, mimicking pathological features of other protein misfolding diseases, including Alzheimer’s and Parkinson’s diseases [18,26].

An intriguing observation was the absence of Aβ plaque deposition in mice treated with both moPrP^Res^ and LPS, despite the presence of significant neuroinflammation. This finding raises the possibility that moPrP^Res^ interferes with LPS-driven amyloidogenic pathways. Previous studies have demonstrated that recombinant prion proteins can bind LPS and assemble into higher-order complexes [5], potentially altering the bioavailability, cellular uptake, or immunological profile of LPS. Such interactions may disrupt the cascade leading to amyloid plaque formation, perhaps by modulating toll-like receptor signaling or inflammasome activation.

Indeed, it is well documented that fibrillar protein aggregates—including those formed by prion and amyloid-β—can activate the NLRP3 inflammasome, driving IL-1β release and amplifying neuroinflammation [27,28,29]. It is conceivable that moPrP^Res^–LPS complexes modulate inflammasome signaling differently from either LPS or misfolded PrP alone, thereby limiting downstream amyloid deposition. Further mechanistic studies are warranted to determine whether these hybrid aggregates exert protective, neutral, or even antagonistic effects within the context of Aβ pathology.

#### 3.5.2. Metabolic Alterations and Obesity

An additional observation is that LPS-only mice developed significant weight gain after ~30 weeks of age—a result consistent with prior reports linking chronic endotoxin exposure to obesity, insulin resistance, and systemic inflammatory dysfunction [24,30]. Adipose tissue, particularly in the abdominal region, may act as a reservoir for pro-inflammatory mediators [24,25], potentially exacerbating neuroinflammation via endocrine or paracrine signaling pathways. Although uncovering the precise mechanism between obesity, neurodegeneration, and inflammation lies beyond the scope of this article, our findings highlight the complex consequences of chronic LPS exposure. Future investigations could clarify whether preventing metabolic derangements or targeting obesity-related inflammation might mitigate LPS-induced neuropathology.

### 3.6. Synergistic Impact of LPS and RML

#### 3.6.1. Acceleration of Classical Prion Disease

The most rapid clinical deterioration and shortest survival times were observed in the RML + LPS co-treatment group, suggesting that systemic inflammation accelerates the clinical progression of prion disease. Surprisingly, histopathological analysis revealed significantly reduced vacuole counts in these animals—particularly in the Cc, Th, Mb, and Cr—when compared to RML-only mice. This paradoxical reduction in spongiform vacuolation was observed in animals that died prior to the study endpoint.

These findings suggest that chronic LPS exposure may accelerate disease onset through mechanisms distinct from classical vacuolar neurodegeneration—potentially involving microglial priming, astroglial reactivity, or altered prion trafficking and neuroimmune signaling. Despite the attenuation of vacuolation at this stage, extensive PrP^Sc^ deposition was still evident in both central nervous system tissues and peripheral lymphoid organs.

This aligns with previous evidence that systemic inflammation promotes prion dissemination via the lymphoreticular system [8,12,16,21,31]. Consistently, PrP^Sc^ was detectable in the spleens of RML + LPS-treated mice as early as 11 weeks post-infection, supporting the notion that peripheral immune tissues may serve as early replication reservoirs.

#### 3.6.2. Lack of Synergism Between LPS and Recombinant Prion Protein

In contrast to the RML + LPS group, LPS co-administration did not exacerbate clinical signs, mortality, or neuropathology in mice treated with moPrP^Res^, which remained devoid of detectable PrP^Sc^ by all tested assays. Notably, vacuole counts in the moPrP^Res^ + LPS group were not increased compared to moPrP^Res^ alone; in fact, LPS co-treatment was associated with slightly lower vacuolation in several brain regions, including the cerebellum and thalamus. This pattern mirrors the paradoxical suppressive effect observed in RML + LPS animals, suggesting that LPS-induced immune modulation may constrain prion-related neurodegeneration in certain contexts. These observations underscore the complexity of prion biology and the influential role of co-factors embedded in brain-derived scrapie isolates (i.e., RML) [3,4]. Whereas chronic inflammation can heighten neuronal vulnerability, an “incomplete” or non-replicative prion conformer like moPrP^Res^ may lack key structural or biochemical features—such as seeding competence or transmissibility—needed to engage in synergistic interactions with systemic inflammation. Together, these findings highlight the importance of prion strain composition and host immune state in shaping disease trajectory and neuropathological outcomes [4,12].

#### 3.6.3. Early Detection in the Spleen and Diagnostic Implications

Consistent with previous work demonstrating that spleen homogenates can reveal prion replication ahead of clinical onset [21,22,31], we found that RML-treated animals (with or without LPS) harbored detectable PrP^Sc^ in spleen samples as early as 11 wpi. This reaffirms the spleen as a pivotal site for prion replication and highlights its utility for presymptomatic diagnosis [31]. Notably, the co-presence of LPS amplified PrP^Sc^ accumulation in peripheral tissues, implicating systemic inflammatory responses in accelerating prion replication outside the CNS [12,16].

### 3.7. Mechanistic Insights and Future Directions

#### 3.7.1. Unified Perspectives on Protein Misfolding and Inflammation

A central theme emerging from this study is that inflammation—whether triggered by bacterial products (LPS) or by prion-rich brain homogenates—can dramatically modulate neurodegenerative processes [12,16,32]. Chronic inflammation appears to promote microglial reactivity, alter blood–brain barrier integrity [33,34], and synergize with misfolded proteins to worsen neuronal damage. While the “protein-only” hypothesis posits that prion diseases are primarily driven by PrP^Sc^ conversion [1,3], our data underscore that infection and neurodegeneration are also shaped by systemic immune states, metabolic context, and the biochemical milieu in which misfolded proteins propagate [2,4,12].

#### 3.7.2. Possible Roles of Oligomeric PrP and Alternative Neurotoxic Species

The moPrP^Res^ variant may adopt oligomeric or partially protease-resistant conformations that escape detection by conventional assays, yet exert potent neurotoxic effects—reminiscent of soluble amyloid-β oligomers implicated in Alzheimer’s disease pathogenesis [7,11]. Unlike large fibrillar aggregates, these smaller assemblies may be diffusible, synaptotoxic, and capable of initiating microglial activation and oxidative stress. Detailed biophysical characterization—using techniques such as conformation-dependent immunoassays, electron microscopy, atomic force microscopy, and spectroscopic methods—will be essential to determine whether moPrP^Res^ exists in such toxic intermediate states in vivo. Investigating these structural features could uncover critical insights into the mechanisms of non-infectious prion toxicity and enable the development of targeted therapies aimed at stabilizing benign PrP conformations, blocking oligomerization, or enhancing cellular clearance of pathological species.

#### 3.7.3. Therapeutic and Prophylactic Interventions

The observation that chronic exposure to bacterial endotoxin can independently trigger Alzheimer’s-like neuropathology and accelerate prion disease underscores the potential of targeting systemic inflammation as a therapeutic strategy. Approaches aimed at reducing peripheral endotoxin burden—such as the use of anti-inflammatory agents, endotoxin-binding compounds, or interventions that reinforce the intestinal epithelial barrier—may attenuate LPS translocation, dampen microglial priming, and delay neurodegenerative progression [35,36,37]. In parallel, the unique neurotoxicity induced by moPrP^Res^, in the absence of PrP^Sc^ accumulation, points to the need for interventions that block early conformational transitions or toxic oligomer formation. Therapeutic efforts could include small molecules or antibodies that stabilize native PrP structure, disrupt pathogenic misfolding, or enhance proteostatic mechanisms. Together, these strategies may offer a two-pronged approach—mitigating both inflammatory triggers and intrinsic prion toxicity—to delay or prevent disease onset in prion and prion-like neurodegenerative disorders.

### 3.8. Chronic Inflammation Alters Prion-Induced Vacuolation in a Stage-Dependent Manner

Detailed histopathological analysis across experimental stages—euthanization (11 wpi), clinical termination, and naturally sick animals—revealed complex, stage-dependent vacuole patterns [8,9,10]. As expected, RML-infected mice at the clinical stage exhibited the most extensive vacuolation across all brain regions. However, surprisingly, the addition of LPS in co-treatment with either RML or moPrP^Res^ consistently reduced vacuole counts, particularly in the Th, Mb, and Cr.

This suppressive effect was statistically significant and reproducible in animals that survived to the termination stage, suggesting a paradoxical modulatory role of chronic inflammation on prion-induced neurodegeneration. However, in terminally sick animals, vacuole counts in the RML + LPS and moPrP^Res^ + LPS groups were similar to those in the RML-only group, with no statistically significant differences. These findings suggest that while LPS may delay or modulate the progression of spongiform pathology at earlier stages, its suppressive effects on vacuolation may not persist into the terminal phase of disease.

In the moPrP^Res^ group, vacuolation was moderate, primarily localized to cerebellar and midbrain regions, and appeared unaffected by LPS. Notably, vacuole counts were negligible in all treatment groups euthanized at 11 wpi, underscoring the progressive nature of neurodegeneration and emphasizing the importance of time point selection in neuropathological studies.

Collectively, these results challenge the assumption that systemic inflammation uniformly amplifies prion pathology. Instead, they suggest that prolonged LPS exposure may precondition neuroimmune responses or disrupt prion trafficking, reducing vacuolar degeneration under certain conditions.

## 4. Study Limitations

One important limitation of this study is the variable and, in some cases, low number of animals per treatment group at later timepoints, particularly at the termination stage (110 wpi), where survival was limited in several groups. In some instances, only one or two animals per group remained, which reduced the statistical power to detect significant differences and restricted our ability to generalize findings at this stage. While we report these data transparently and include them for completeness, interpretations from groups with low replications should be considered descriptive rather than conclusive. Future studies with larger group sizes and survival-balanced experimental designs will be essential to validate and extend the findings presented here.

Another limitation of this study is that while we observed prion-like neuropathological changes induced by moPrP^Res^ and LPS, we did not include full in vivo transmission results within this manuscript. Although we conducted intracerebral and oral infectivity experiments using purified moPrP^Res^ and LPS to assess their transmissibility, these findings will be reported separately due to their distinct methodological design and extended observation periods. As a result, the current study does not evaluate long-term infectivity outcomes such as clinical disease onset or secondary transmission. Therefore, our use of the term “prion-like” is confined to describing histological, immunohistochemical, and behavioral features resembling prion disease, without implying definitive infectivity. Further work is ongoing to fully characterize the transmissibility of these prion-like conformers in vivo.

## 5. Conclusions

This study provides new insights into the interplay between inflammation and protein misfolding in the pathogenesis of prion diseases. We demonstrate that recombinant moPrP^Res^, generated in vitro in the presence of LPS and lacking classical protease-resistant PrP^Sc^, can still induce neurodegeneration in wild-type mice, although with regionally confined effects. Furthermore, chronic subcutaneous exposure to LPS alone produced moderate spongiform changes, particularly in the cerebellum and midbrain, suggesting its potential utility as a model for endotoxin-induced, Alzheimer’s-like neurodegeneration. Intriguingly, rather than amplifying disease severity, systemic LPS co-treatment suppressed vacuolation in prion-infected mice, indicating a modulatory role of inflammation that may interfere with prion replication or mitigate neurotoxicity. Analysis of subclinical animals euthanized at 11 weeks revealed minimal vacuolation, underscoring the importance of temporal dynamics and the necessity of late-stage assessments to capture the full extent of prion-associated neuropathology. Collectively, these findings indicate the need for a more integrative understanding of prion pathogenesis—one that accounts for the systemic inflammatory milieu, prion strain-specific features, and host neuroimmune interactions—and point toward therapeutic strategies that target host–pathogen crosstalk rather than prion replication alone.

## 6. Materials and Methods

### 6.1. Ethical Approval

This study was conducted in accordance with institutional and national ethical standards. Approval was obtained from the University of Alberta Animal Care and Use Committee for Health Sciences Laboratory Animal Services. All procedures adhered to the guidelines of the Canadian Council on Animal Care [38], and every aspect of animal handling conformed to the University of Alberta’s policies on animal welfare. The well-being of the experimental animals was prioritized throughout the course of the study.

### 6.2. Experimental Design and Animals

A total of 90 wild-type female FVB/N mice (5 weeks old) were obtained from Charles River Laboratories (Wilmington, MA, USA). Mice were randomly assigned to six treatment groups (n = 15 per group): saline (negative control), lipopolysaccharide (LPS) from *Escherichia coli* O111:B4, moPrP^Res^ (amino acids 29–232) incubated with *E. coli* O111:B4 LPS (Sigma-Aldrich, St. Louis, MO, USA), moPrP^Res^ + LPS, RML + LPS, and RML alone (positive control).

Bacterial LPS and saline were delivered continuously via subcutaneously implanted ALZET^®^ osmotic mini-pumps (Cupertino, CA, USA), with a flow rate of 0.11 µL/h over six weeks. The LPS dosage was standardized at 0.1 µg per gram of body weight. Simultaneously, a single subcutaneous injection of either moPrP^Res^ (45 µg per mouse) or the RML prion strain (containing 10^7^ ID_50_ units of scrapie prions) was administered at the time of pump implantation. The moPrP^Res^ (amino acids 29–232) was kindly provided by Dr. David Wishart’s laboratory (University of Alberta) and injected in a total volume of 200 µL per mouse. For further experimental design details, refer to Hailemariam et al. [6].

### 6.3. Weight Measurement

To monitor health status and physiological development, body weights were recorded for each mouse beginning at six weeks of age, coinciding with the start of treatment. Measurements were performed monthly. As the study progressed and mortality increased, surviving mice were additionally weighed at 102 and 110 weeks of age to assess the long-term effects of treatment.

### 6.4. Mouse Recombinant Prion Protein

Lyophilized LPS from *Escherichia coli* O111:B4 (Sigma-Aldrich, St. Louis, MO, USA) was reconstituted in Milli-Q double-distilled water (ddH_2_O) to a working concentration of 5 mg/mL. This LPS stock solution was then used to dissolve lyophilized mouse recombinant prion protein (moPrP, residues 29–232) to a final concentration of approximately 0.5 mg/mL, yielding a 1:1 weight ratio of moPrP:LPS. Given that the average molecular weight of LPS is ~10 kDa—approximately half that of moPrP (∼20–22 kDa)—this weight ratio corresponds to a molar ratio of approximately 2:1. Due to the undefined and heterogeneous molecular structure of LPS, all experimental ratios were maintained on a mass (mg) basis for consistency and comparability.

Structural conversion of moPrP was monitored by circular dichroism (CD) spectroscopy in the far-UV range (190–260 nm) using an Olis DSM 17 spectropolarimeter (Bogart, GA, USA). Spectra were acquired at 25 °C in a 0.02 cm path-length quartz cuvette, with five consecutive scans averaged per sample. Protein concentrations were standardized at 0.5 mg/mL (approximately 25 µM). Baseline spectra (reference buffer only) were subtracted from the sample spectra before calculating molar ellipticity. An average amino acid molecular weight of 113.64 g/mol was used for calculations. Secondary structural content was determined using the CDPro software (v 4.31) suite with the CONTINLL algorithm and the SP22X reference set. Conversion endpoints were defined as follows: a β-sheet content >25% with helical content <15% indicated β-sheet conversion, while a β-sheet content >30% with helical content <10% indicated fibrillar aggregation.

Following conversion, residual LPS was removed using polymyxin B agarose resin (Sigma-Aldrich). Briefly, 500 µL of resin was added to a 1.5 mL microcentrifuge tube and equilibrated with three washes of 500 µL of ammonium bicarbonate buffer (100 mM, pH 8.0). Subsequently, 250 µL of the moPrP/LPS solution was added and incubated at room temperature for 60 min to allow LPS binding. The resin was pelleted by centrifugation at 850× *g* for 5 min, and the supernatant—containing the LPS-depleted moPrP^Res^—was collected. This LPS-removal step was repeated three additional times with fresh equilibrated resin. The final supernatant was assessed for residual endotoxin using the Pyrochrome Limulus Amebocyte Lysate (LAL) assay (Associates of Cape Cod Inc., East Falmouth, MA, USA), and structural confirmation of moPrP^Res^ was re-evaluated by CD spectroscopy.

### 6.5. Euthanasia

Euthanasia was performed at two key time points: (1) 11 weeks post-infection (wpi), and (2) the terminal stage of disease. At 11 wpi, five mice from each treatment group were euthanized. These animals exhibited normal body weight and showed no clinical signs of prion disease or other abnormalities. The remaining ten mice per group were monitored until the terminal stage, which was defined by the appearance of progressive neurological signs including kyphosis, ataxia, dysmetria, tremors, head tilt, tail rigidity, circling behavior, bradykinesia, proprioceptive deficits, stupor, loss of deep pain sensation, diminished grooming, and marked body weight loss.

### 6.6. Tissue Preparation

Prior to euthanasia, mice were anesthetized with isoflurane gas, and the absence of reflexes was confirmed to ensure complete analgesia. Euthanasia was performed by cardiac puncture, during which total blood volume was collected. Immediately thereafter, brains were harvested and bisected sagittally. One hemibrain was fixed in 10% neutral buffered formalin phosphate (Sigma-Aldrich, St. Louis, MO, USA) using a volume ten times that of the tissue and incubated for at least 48 h at room temperature. Following fixation, tissues were rinsed under tap water for 20 min and transferred to 15 mL tubes containing 70% ethanol (Commercial Alcohol, Winnipeg, MB, Canada) for storage at 4 °C until further processing.

Brain tissues were processed for immunohistochemical (IHC) analysis to evaluate the regional distribution and intensity of disease-associated prion protein (PrPSc), amyloid-beta (Aβ) plaque deposition, astrogliosis (via GFAP staining), and spongiform vacuolation.

### 6.7. Hematoxylin and Eosin Staining

Brain tissues were processed for hematoxylin and eosin (H&E) staining according to the protocol described by Chishti et al. [39]. Formalin-fixed tissues were embedded in paraffin (Formula “R” paraffin, Surgipath^®^, Leica Biosystems, Nussloch, Germany) and sectioned coronally. Sections were mounted on adhesive-coated glass slides and dried overnight at 37 °C. Deparaffinization was performed using xylene followed by rehydration through a graded ethanol series and deionized water. Slides were stained with filtered Mayer’s hematoxylin (Fisher Scientific, Waltham, MA, USA), counterstained with eosin Y, and dehydrated. Digital images of stained sections were captured using the NanoZoomer XR digital slide scanner (Hamamatsu Photonics, Shizuoka, Japan).

### 6.8. Vacuole Quantification

Spongiform vacuoles were assessed in H&E-stained coronal brain sections scanned at 40× magnification using the Hamamatsu NanoZoomer S360 Digital Slide Scanner, Hamamatsu Photonics, Bridgewater, New Jersey, USA. Whole-slide images were analyzed using NDP.view2 software (version U12388-01; Hamamatsu Photonics, Bridgewater, New Jersey, USA). Regions of interest—including the cerebral cortex (Cr), thalamus (Th), midbrain (Mb), and cerebellum (Cr)—were digitally magnified to an effective magnification of 5.5×. Built-in contrast and clarity enhancement tools were applied to optimize visualization of vacuoles without introducing artificial artifacts.

Vacuoles were defined as discrete, round or oval optically clear structures measuring ≥5 µm in diameter, and were carefully distinguished from perivascular spaces, fixation artifacts, and tissue tears. For each mouse, three non-overlapping high-power fields (HPFs) were selected per brain region. Manual vacuole counts were performed using NDP.view2 software (version U12388-01; Hamamatsu Photonics, Bridgewater, NJ, USA) by two independent observers blinded to treatment groups. The mean number of vacuoles per HPF was calculated for each region per animal. Group means ± standard error of the mean (SEM) were computed and used for statistical analyses. Inter-observer agreement exceeded 95%, and any discrepancies were resolved through consensus-based review.

### 6.9. PrP^Sc^ Immunohistochemical Staining

Detection of disease-associated prion protein (PrP^Sc^) was performed following the protocol described by Bell et al. [40], optimized to achieve maximal clearance of native cellular prion protein (PrP^C^). Brain sections were sequentially treated with 98% formic acid, 4 M guanidine thiocyanate, and 3% hydrogen peroxide (H_2_O_2_) to denature PrP^C^ and expose disease-specific epitopes. Immunostaining was carried out using a mouse monoclonal SAF83 antibody (1:500 dilution; Cayman Chemical, Ann Arbor, MI, USA), followed by incubation with a streptavidin-conjugated peroxidase. The immunoreaction was visualized using a diaminobenzidine (DAB) substrate (Vector Laboratories, Burlingame, CA, USA), and slides were counterstained with Mayer’s hematoxylin (Fisher Scientific, Waltham, MA, USA) for nuclear contrast.

### 6.10. Amyloid Plaque Staining

Amyloid-beta (Aβ) plaque detection was performed using thioflavine S staining as described by Chishti et al. [39]. Formalin-fixed, paraffin-embedded brain sections were incubated in a 1% aqueous solution of thioflavine S (Sigma-Aldrich, St. Louis, MO, USA), a fluorescent dye with high specificity for beta-sheet-rich amyloid fibrils. After incubation, slides were differentiated and dehydrated through a graded ethanol series. Slides were then dried in the dark and examined for amyloid plaque (Aβ) deposition using a Nikon Eclipse 90i fluorescence microscope (Nikon Instruments Inc., Melville, NY, USA).

### 6.11. Immunohistochemical Detection of Astrogliosis

Astrogliosis was assessed by immunohistochemical staining for glial fibrillary acidic protein (GFAP), following the methodology of Chishti et al. [39]. After deparaffinization and rehydration, brain sections were incubated with an anti-GFAP primary antibody (Sigma-Aldrich, St. Louis, MO, USA; BD Pharmingen, Mississauga, ON, Canada). This was followed by incubation with streptavidin-peroxidase (Invitrogen, Carlsbad, CA, USA) for 16 min. Detection was achieved using DAB (i.e., 3,3′-Diaminobenzidine) chromogen (BD Pharmingen, Mississauga, ON, Canada), developed for up to 20 min to yield a brown precipitate marking GFAP-positive astrocytes. Slides were counterstained with Mayer’s hematoxylin (Thermo Fisher Scientific, Waltham, MA, USA), cleared with xylene (Sigma-Aldrich, Sigma-Aldrich, St. Louis, MO, USA), and mounted with Cytoseal 60 (Thermo Fisher Scientific, Waltham, MA, USA). Slides were then left to dry at room temperature for 48 h before imaging.

### 6.12. Western Blot Assay

Brain and spleen tissues were processed into 10% (*w/v*) homogenates in 1× phosphate-buffered saline (PBS; Bio-Rad, Hercules, CA, USA) under sterile conditions. Total protein concentration was determined using the bicinchoninic acid (BCA) protein assay kit (Thermo Fisher Scientific, Waltham, MA, USA). For downstream analysis, 250 µg of total protein from brain samples and 400 µg from spleen samples were used.

Each homogenate was diluted in 250 µL of radioimmunoprecipitation assay (RIPA) buffer (Sigma-Aldrich, St. Louis, MO, USA). Proteinase K (PK; 50 µg/mL final concentration) was added to each sample to digest non-resistant PrP^C^ isoforms. To aid visualization, 2 µL of 0.02% bromophenol blue (Bio-Rad, Hercules, CA, USA) was added to each sample. Samples were briefly vortexed and incubated at 37 °C for 1 h. Proteolysis was halted by the addition of 25 µL phenylmethylsulfonyl fluoride (PMSF; Sigma-Aldrich, St. Louis, MO, USA) to a final concentration of 5 mM. Samples were then incubated at room temperature for 5 min and centrifuged at 20,000× *g* for 60 min at 4 °C. The supernatant was discarded, and the resulting pellet was resuspended in 15 µL of 2× sample buffer (SB) and boiled for 10 min to denature proteins.

Protein samples (20–25 µL) were loaded into wells of NuPAGE^®^ Bis-Tris 4–12% gradient mini gels (Invitrogen, Carlsbad, CA, USA) and separated via SDS-PAGE at 200 V for 50 min. Precision Plus Protein™ WesternC chemiluminescent molecular weight markers (Bio-Rad, Hercules, CA, USA) were included in each gel.

Proteins were transferred onto PVDF (i.e., polyvinylidene difluoride) membranes overnight using a wet transfer system (Bio-Rad, Hercules, CA, USA) at 20 V. Following transfer, membranes were briefly rinsed with 1× TBS-T (0.5% Tween-20; Sigma-Aldrich) and incubated overnight at 4 °C with the monoclonal anti-prion protein antibody Sha31 (1:30,000; Bertin Pharma, Montigny-le-Bretonneux, France) diluted in 1× TBS-T (0.5%).

The following day, membranes were washed and incubated for 1 h at room temperature with goat anti-mouse horseradish peroxidase (HRP)-conjugated secondary antibody (1:10,000; Bio-Rad, Hercules, CA, USA) in 1× TBS-T (0.1%) supplemented with 5% non-fat dry milk (Carnation, Smucker Foods of Canada, Markham, ON, Canada). After final washes, signal detection was performed using Pierce^®^ ECL Plus Western blotting Substrate (Thermo Fisher Scientific, Waltham, MA, USA), and bands were visualized with the ImageQuant LAS 4000 digital imaging system (GE Healthcare Life Sciences, Quebec, QC, Canada).

### 6.13. Scrapie Cell Assay

The scrapie cell assay was performed based on the standard protocol described by Mahal et al. [25], with minor adaptations. L929 mouse fibroblast cells (ATCC, Manassas, VA, USA) were used as the permissive cell line for prion infection. Brain homogenates (10% *w*/*v* in 1× PBS) were prepared from various treatment groups, including saline (negative control), *Escherichia coli* O111:B4 LPS, moPrP^Res^, moPrP^Res^ + LPS, RML + LPS, and RML (positive control). Thirty microliters of each homogenate were aliquoted into wells of a 96-well tissue culture plate (Corning Costar, Tewksbury, MA, USA) and serially diluted (0.1% to 0.0001%) in six replicates.

A suspension of L929 cells (~5000 cells in 20 µL) was added to each well. Plates were incubated for 3–5 min at 37 °C before the addition of 150 µL Dulbecco’s Modified Eagle Medium (DMEM) supplemented with 10% horse serum (Sigma-Aldrich, St. Louis, MO, USA). The plates were then incubated at 37 °C in a humidified atmosphere with 5% CO_2_ for five days. Cells were passaged twice (1:4 and 1:7 dilutions), with a five-day incubation period between each passage to allow prion propagation.

For prion detection, an ELISPOT-based assay was performed. Ninety-six-well ELISPOT plates (Millipore, Billerica, MA, USA) were pre-activated by adding 60 µL of 70% ethanol per well for 3 min, followed by three washes with 1× TBS (Sigma-Aldrich, St. Louis, MO, USA). To maintain hydration of the nitrocellulose membranes, 30 µL of 1× TBS was left in each well before seeding. Subsequently, 20,000 L929 cells were added to each well and allowed to adhere. The plates were subjected to vacuum filtration and dried at 50 °C for one hour.

Cell lysis was achieved by adding 60 µL of Radioimmunoprecipitation assay (RIPA) buffer containing 5 µg/mL proteinase K (PK; Invitrogen, Carlsbad, CA, USA) to each well, followed by a 90 min incubation at 37 °C. After lysis, the wells were washed three times with 1× TBS. To stop PK activity, 100 µL of 2 mM phenylmethylsulfonyl fluoride (PMSF; Sigma-Aldrich, St. Louis, MO, USA) in TBS was added to each well and incubated on a 3D rotator (Model 4631, Thermo Scientific, Waltham, MA, USA) for 10 min at room temperature. The PMSF solution was then removed, and the wells were washed three times with TBS.

Next, 100 µL of 3 M guanidine thiocyanate (GdnSCN; Fisher Scientific, Waltham, MA, USA) was added to each well, followed by another 10 min incubation on the rotator at room temperature. After removing the GdnSCN, wells were washed four times with 1× TBS. Blocking was performed by adding 100 µL of 5% skim milk (prepared in TBS; Carnation^®^, Smucker Foods of Canada, Markham, ON, Canada) to each well, with incubation at room temperature for 1 h.

Following blocking, 100 µL of SAF83 monoclonal anti-PrP antibody (1:1000 dilution in TBS; Cayman Chemical, Ann Arbor, MI, USA) was added and incubated for 2 h at room temperature on the rotator. After antibody removal and three washes with TBS, 100 µL of goat anti-mouse alkaline phosphatase-conjugated secondary antibody (1:5000; Bio-Rad, Hercules, CA, USA) was added and incubated for 90 min at room temperature with rocking.

After washing three times with TBS, 60 µL of alkaline phosphatase buffer (100 mM Tris-HCl, 100 mM NaCl, 5 mM MgCl_2_·6H_2_O; Sigma-Aldrich) was added to each well and incubated for 10 min. This was followed by the addition of 60 µL of BCIP/NBT substrate solution (Promega, Madison, WI, USA) and a 20 min incubation for color development. The wells were then washed four times with distilled water, and the plates were allowed to dry overnight in the dark.

### 6.14. Statistics

Body weight trends were analyzed using the MIXED procedure in SAS version 9.3 (SAS Institute Inc., Cary, NC, USA). The following linear mixed model was applied:

Y_ijkl_ = μ + t_i_ + p_j_ + (tp)_ij_

where

Y_ijkl_ = observed dependent variable (body weight),

μ = overall population mean,

t_i_ = fixed effect of treatment group,

p_j_ = fixed effect of time (period, i.e., age in weeks),

(tp)_ij_ = fixed interaction effect between treatment and period,

ε_ijkl_ = residual error, assumed to be normally distributed with mean zero and constant variance.

Degrees of freedom were estimated using the Kenward–Roger adjustment. Least squares means (LS-means) were compared using the PDIFF option in SAS, and statistical significance was declared at *p* < 0.05.

In addition, survival analysis was conducted using GraphPad Prism (version X; GraphPad Software Inc., San Diego, CA, USA). Survival curves were generated using the Kaplan–Meier method, and differences between treatment groups were evaluated using the log-rank (Mantel–Cox) test.

#### Statistical Analysis of Vacuole Counts

Spongiform vacuolation was quantified in hematoxylin and eosin (H&E)-stained sagittal brain sections scanned using a Hamamatsu NanoZoomer whole-slide scanner. Digital files were opened and visualized using Hamamatsu NDP.view2 software (version U12388-01; Hamamatsu Photonics, Bridgewater, New Jersey, USA), where images were magnified to 5.5× for analysis. Four anatomically defined brain regions were evaluated: the Cc, Th, Mb, and Cr.

For each animal, one representative brain section was selected. Vacuole counts were performed within a fixed analysis area of approximately 1.0 mm^2^ per region, centered on neuroanatomical landmarks to ensure consistency across animals and treatment groups. Regions were manually delineated and adjusted to avoid artifacts or damaged tissue.

Image preprocessing was conducted using ImageJ (ver. 1.51; Fiji distribution, The Eliceiri/LOCI lab, WI, USA) to improve visual clarity and counting accuracy. A standardized filtering sequence was applied: (1) conversion to 8-bit grayscale, (2) background subtraction with a rolling ball radius of 50 pixels, (3) Gaussian blur (σ = 1.0), and (4) manual thresholding. Only vacuoles with a diameter greater than 5 μm were considered for counting. Annotation and quantification were performed manually by a single observer blinded to treatment groups.

Data are reported as mean vacuole count ± standard error of the mean (SEM) for each brain region and experimental group. One-way analysis of variance (ANOVA) followed by Tukey’s post hoc test was used to assess statistical differences between groups within each brain region. A threshold of *p* < 0.05 was considered statistically significant. Results are presented in the main text, with quantitative data summarized descriptively within the Section 2.

## Data Availability

All data generated in this study are presented within this published article.
